# Synthesis of P- and N-doped carbon catalysts for the oxygen reduction reaction via controlled phosphoric acid treatment of folic acid

**DOI:** 10.3762/bjnano.10.148

**Published:** 2019-07-25

**Authors:** Rieko Kobayashi, Takafumi Ishii, Yasuo Imashiro, Jun-ichi Ozaki

**Affiliations:** 1 Graduate School of Science and Technology, Gunma University, 1-5-1 Tenjin-cho, Kiryu, Gunma 376-8515, Japan; 2 R&D Center, Nissinbo Holdings Inc., 1-2-3 Onodai, Midori-ku, Chiba 267-0056, Japan; 3 Business Development Division, Nisshinbo Holdings Inc., 2-31-11, Nihonbashi Ningyo-cho, Chuo-ku, Tokyo 103-8650, Japan

**Keywords:** folic acid, oxygen reduction reaction, phosphoric acid treatment, PN-doped carbon catalysts, polymer electrolyte fuel cells

## Abstract

Herein, we synthesized P- and N-doped carbon materials (PN-doped carbon materials) through controlled phosphoric acid treatment (CPAT) of folic acid (FA) and probed their ability to catalyze the oxygen reduction reaction (ORR) at the cathode of a fuel cell. Precursors obtained by heating FA in the presence of phosphoric acid at temperatures of 400–1000 °C were further annealed at 1000 °C to afford PN-doped carbon materials. The extent of precursor P doping was maximized at 700 °C, and the use of higher temperatures resulted in activation and increased porosity rather than in increased P content. The P/C atomic ratios of PN-doped carbon materials correlated well with those of the precursors, which indicated that CPAT is well suited for the preparation of PN-doped carbon materials. The carbon material prepared using a CPAT temperature of 700 °C exhibited the highest ORR activity and was shown to contain –C–PO_2_ and –C–PO_3_ moieties as the major P species and pyridinic N as the major N species. Moreover, no N–P bonds were detected. It was concluded that the presence of –C–PO_2_ and –C–PO_3_ units decreases the work function and thus raises the Fermi level above the standard O_2_/H_2_O reduction potential, which resulted in enhanced ORR activity. Finally, CPAT was concluded to be applicable to the synthesis of PN-doped carbon materials from N-containing organic compounds other than FA.

## Introduction

The widespread application of fuel cells as clean energy sources is the most desirable way of realizing a low-CO_2_-emission society. In conventional polymer electrolyte fuel cells (PEFCs), both anode and cathode reactions are catalyzed by Pt. Compared to the anode reaction, the cathode reaction, namely the oxygen reduction reaction (ORR), is rather slow and hence requires the use of larger amounts of Pt [1], which increases the cost of PEFCs and prevents their wide application as domestic, back-up, and vehicle power sources. The cost of cathode catalysts can be reduced in a number of ways, e.g., by alloying Pt with base metals [2], forming core–shell particles with base-metal cores covered by thin Pt layers [3], and developing non-Pt catalysts. In particular, the implementation of non-Pt or non-precious-metal cathode catalysts is the ultimate goal of PEFC development.

Since the discovery of the ORR activity of cobalt phthalocyanine in 1964 [4], numerous studies have focused on the synthesis of non-precious metal ORR catalysts such as those based on carbon [5]. The thermal treatment of carbon materials impregnated with N_4_–M complexes was found to afford highly active and durable ORR catalysts. Since then, much effort has been directed at clarifying the nature of the active sites in these catalysts and the ORR activity has been predominantly ascribed to N*_x_*–M (M = Co, Fe) moieties on the surface of the carbon supports [6–7]. Our research group has identified and characterized different types of non-Pt ORR catalysts, the so-called carbon alloy catalysts (CACs) [8]. We prepared two types of CACs (nanoshell-containing carbon materials [9–10] and BN-doped carbon materials [11]) and further improved their ORR activity and durability to afford a commercial CAC [12–13] and thus realized the world’s first portable PEFC cell containing a non-precious-metal cathode catalyst [14–15].

Much effort has been directed at the development of transition-metal-free carbon catalysts for the ORR, with the best practical performance so far observed for N-doped carbon materials [16]. For example, a recently reported metal-free catalyst based on N-doped carbon nanotubes showed high ORR activity even under acidic conditions and allowed for facile electricity generation when employed as a single-cell cathode [17]. The ORR activity of carbon-based catalysts can be substantially improved by their simultaneous doping with N and other elements. In 2007, we reported that carbon prepared by carbonization of a N- and B-doped furan resin exhibited an increased ORR activity in sulfuric acid solution [11], and since then, much attention has been directed at the activation of carbon catalysts through co-doping [18]. The concept of co-doping has been even extended to three-component catalysts, as exemplified by studies on N, P, S-doped and N, P, F-doped carbon materials [19–20]. Strelko et al. used theoretical methods to establish an interesting relationship between the bandgap energy of a given catalyst and its ability to promote reactions involving electron transfer [21]. Moreover, P-doping of graphitic layers was revealed to have an effect similar to that of N-doping and hence, co-doping with P and N was found to be an effective way of increasing the ORR activity of carbon materials [22–26]. Most of the reported PN-doping techniques involve the carbonization of [N-containing polymer + P-containing compound] mixtures or of ionic liquids containing both N and P, i.e., employ special compounds or their combinations as starting materials.

Herein, to establish a more generalized PN-doping method allowing for the use of more common compounds, we developed the technique of controlled phosphoric acid treatment (CPAT) that is potentially applicable to non-special N-containing organic compounds and applied it to folic acid (FA) as a commonly occurring N-containing organic compound. During CPAT, phosphoric acid (PA) acts as both a P-doping agent [20,27–31] and a chemical activator to introduce pores [32–33]. The CPAT method, we used here, includes pretreatment with phosphoric acid at various temperatures to alter the properties of the precursors of carbon materials. In the present study, PN-doped precursors synthesized at CPAT temperatures of 400–800 °C were carbonized at 1000 °C to prepare PN-doped carbon materials, and factors influencing the ORR catalytic activity of these carbon materials were investigated in detail.

## Results

### Structure, chemical composition and ORR activity of precursors

The CPAT temperature affected both the BET specific surface area (BET-SSA) and surface elemental composition of the precursors, as exemplified by values derived from X-ray photoelectron spectra of P-series precursors (Table 1, for the naming scheme of the samples see section “Experimental”). The N_2_ adsorption isotherms together with the micropore size distribution curves are given in Figure S1 (Supporting Information File 1). The BET specific surface area values were calculated from these isotherms. The BET-SSAs of samples prepared at CPAT temperatures below 700 °C were estimated to be of several square meters per gram but rapidly increased at CPAT temperatures above 800 °C, with maximum values obtained at 1000 °C. This behavior was different from that of H-series precursors.

**Table 1:** Surface properties of H- and P-type precursors (for the naming scheme of the samples see section “Experimental”).

sample	BET-SSA (m^2^·g^−1^)	surface composition			

		C (atom %)	N/C	O/C	P/C

H-400	20	61.7	0.206	0.415	—
H-500	65	67.2	0.243	0.246	—
H-600	115	61.4	0.213	0.415	—
H-700	56	79.2	0.091	0.172	—

H-1000	84	81.5	0.055	0.172	—

P-400	2	76.0	0.046	0.258	0.011
P-500	7	72.6	0.059	0.292	0.027
P-600	8	70.2	0.088	0.286	0.051
P-700	6	50.0	0.143	0.702	0.153
P-800	277	68.2	0.095	0.296	0.074

P-1000	1014	84.0	0.043	0.125	0.022

**Figure 1:**
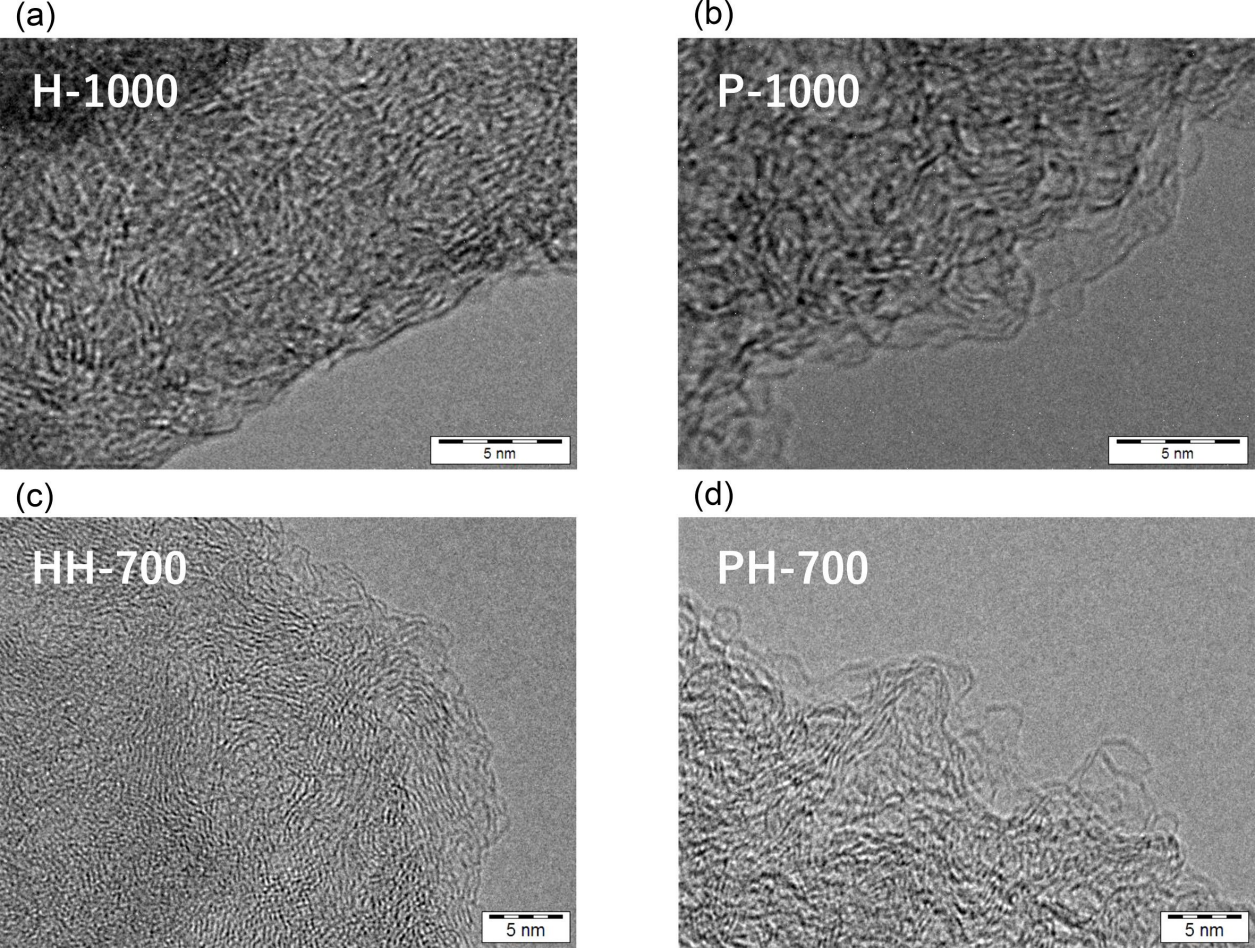
TEM images of the carbon materials. (a) H-1000, (b) P-1000, (c) HH-700, (d) PH700.

Figure 1a,b shows the transmission electron microscopy (TEM) images of the selected samples. Comparing the images of (a) H-1000 and (b) P-1000 revealed differences in the carbon structure. P-1000 is less dense than H-1000 and exhibits a round surface composed of graphitic layers.

**Figure 2:**
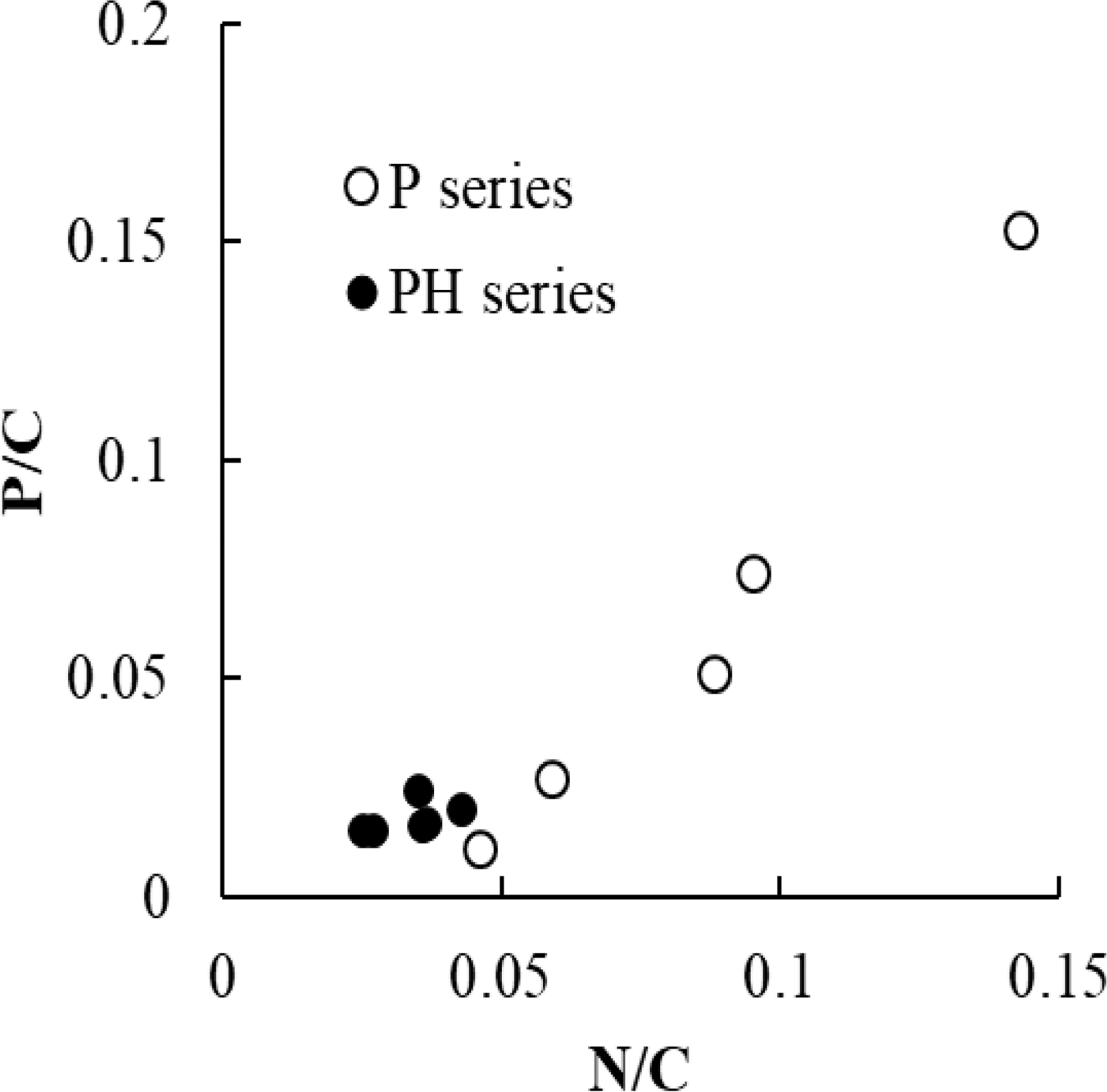
Correlations between P/C atomic ratio and N/C atomic ratio of P-series precursors (open circles) and HP-series carbon materials (closed circles).

The results of X-ray photoelectron spectroscopy (XPS) analysis demonstrated that when pretreatment was performed in the absence of PA, the N content of the carbon materials decreased with increasing temperature. On the other hand, in the presence of PA, the N/C atomic ratio initially increased with increasing CPAT temperature, reaching a maximum at 700 °C, and then decreased again. The O/C ratio behaved similarly to the N/C ratio regardless of the presence of PA but could not be accurately estimated because of the effects of atmospheric moisture and oxygen. The P/C ratio of P-series precursors was maximal at a CPAT temperature of 700 °C, i.e., it behaved similarly to the N/C ratio. Thus, CPAT promoted both the development of specific surface area and P doping, and the relative contributions of these roles were determined by temperature, i.e., P-doping was dominant below 700 °C, while chemical activation was dominant at higher temperatures [34]. Figure 2 shows the correlation between P/C ratio and N/C ratio in P-series precursors and HP-series carbon materials. It indicates the presence of some chemical interactions between the nitrogen in folic acid and the phosphorus in phosphoric acid, which will be discussed in the “Discussion” section.

**Figure 3:**
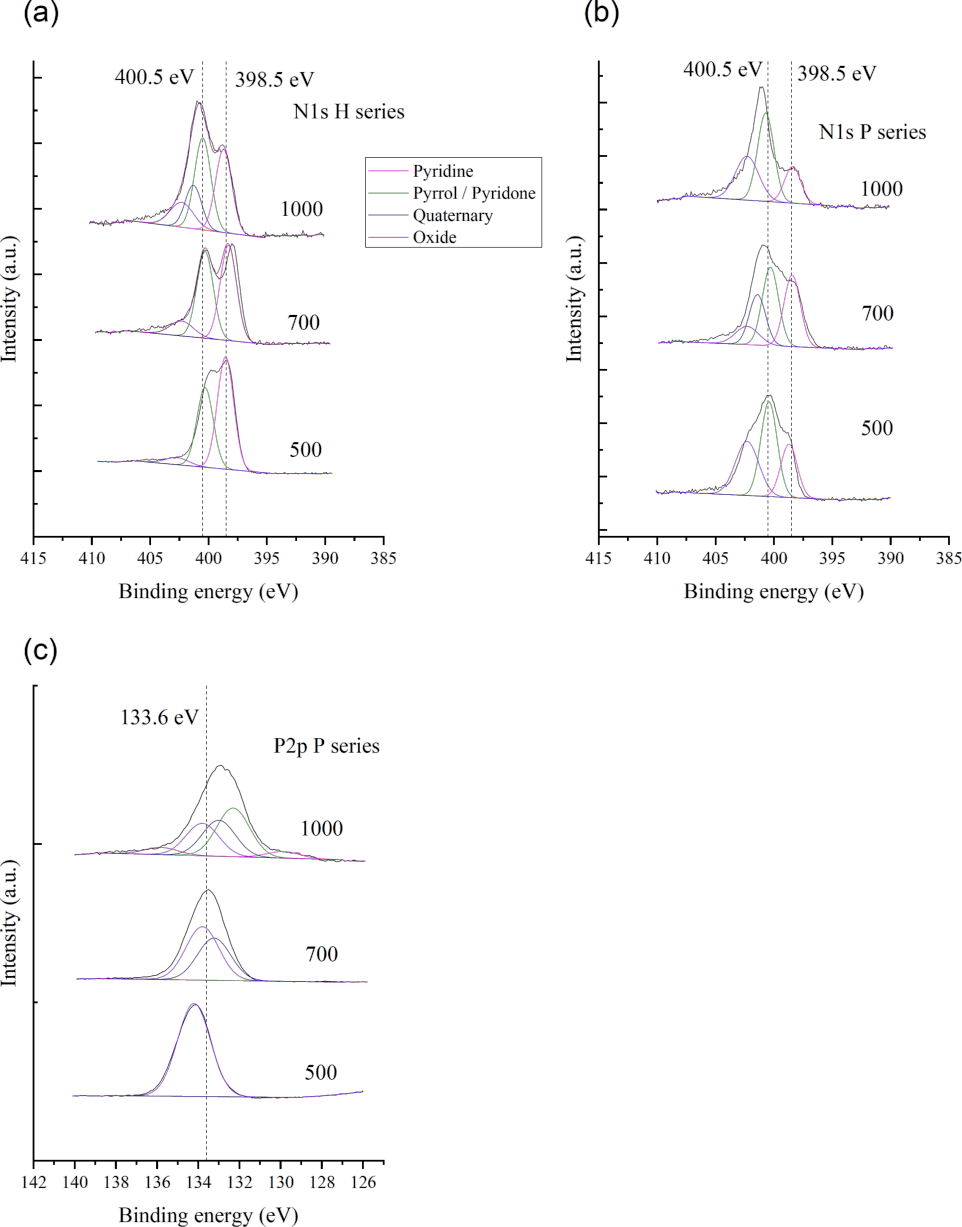
N 1s spectra of (a) H-series and (b) P-series precursors. (c) P 2p spectra of P-series precursors. Results of deconvolution are also presented.

The chemical states of N in P-series precursors were studied by XPS (Figure 3a,b), which revealed that the shapes of N 1s spectra depended on the pretreatment temperature and the presence/absence of PA. H-series precursors featured N 1s spectra with two peaks, the positions of which were affected by CPAT temperature (Figure 3a). For FA pretreated at 500 °C (H-500), these peaks were located at 398.5 and 400.0 eV, while for H-700, peaks at 397.9 and 400.5 eV were observed, and for H-1000, signals were detected at 398.5 and 401.1 eV. The broad N 1s spectra (Figure 3b) of P-series precursors prepared at 500 and 700 °C were assumed to be a superposition of several peaks; the results of its deconvolution are also presented in Figure 3a,b. For example, the spectrum of P-500 was deconvoluted into peaks at 398.5, 400.5, and 402.5 eV, while that of P-700 was deconvoluted into peaks at 398.5, 399.8, and 401.0 eV. In contrast, the N 1s spectrum of P-1000 featured two overlapping peaks centered at 398.5 and 401.7 eV.

Conventionally, peaks at 398.5, 400.5, 401, and 402 eV in the N 1s spectra of N-doped carbon materials are assigned to pyridinic, pyrrole/pyridone-type, quaternary, and oxygen-bonded (oxidized) N, respectively (Table 2). Thus, P-700 contained quaternary N incorporated into graphite layers, as exemplified by the corresponding peaks at 401–400.7 eV. The peak of pyridinic N (398.5 eV), clearly observed for H-series precursors, was less pronounced in the case of P-series precursors, e.g., the intensity of this peak was higher for H-1000 than for P-1000.

**Table 2:** Distribution of the N species in H-type and P-type precursors.

sample	N/C	N_pyridine_	N_pyrrol_	N_quaternary_	N_oxides_

H400	0.207	0.43	0.40	0.00	0.18
H500	0.243	0.56	0.39	0.00	0.05
H600	0.214	0.47	0.42	0.00	0.12
H700	0.091	0.47	0.43	0.00	0.10
H1000	0.055	0.33	0.36	0.17	0.14

P400	0.046	0.14	0.53	0.00	0.33
P500	0.059	0.33	0.42	0.00	0.25
P600	0.088	0.44	0.40	0.00	0.16
P700	0.143	0.32	0.35	0.22	0.12
P800	0.095	0.34	0.32	0.21	0.13
P1000	0.043	0.12	0.47	0.00	0.33

The P 2p XPS spectra of the P-series precursors are presented in Figure 3c. The peak shifted from 134.2 to 133.0 eV with the increase of the CPAT temperature. The figure also includes the results of peak deconvolution by assuming the presences of the five species given in the legend. The P-species varied with the CPAT temperature.

The ORR voltammograms of the precursors are presented in Figure S4 (Supporting Information File 1). Both H-series and P-series precursors showed increased ORR activity with the CPAT temperature. The temperature-dependence of ORR activity is remarkably large for P-series precursors. The highest ORR activity among the precursors was achieved by P-1000.

### Structure, chemical composition, and electronic properties of carbonized FA

PH-series carbon materials were prepared by thoroughly rinsing P-series precursors with water to remove excess PA and subjecting them to carbonization at 1000 °C. The same operation was also performed for H-series precursors to afford HH-series carbon materials. The N_2_ adsorption isotherms are presented in Figure S5 (Supporting Information File 1) with the micropore size distribution calculated by the MP-method. The BET-SSAs of these two carbon series exhibited different behaviors (Table 3), e.g., those of HH-series carbon materials were almost constant (ca. 30 m^2^·g^−1^) even though the corresponding precursors showed different BET-SSA values, whereas the BET-SSA of PH-series carbon materials increased with increasing CPAT temperature. Specifically, the samples with CPAT temperature above 700 °C showed remarkable increases of BET-SSA. This might be caused by desorption or destruction of instable compounds formed by CPAT at these temperatures.

**Table 3:** BET-SSAs and XPS-determined elemental surface compositions of HH- and PH-series carbon materials.

sample	BET-SSA (m^2^·g^−1^)	surface composition			

		C (atom %)	N/C	O/C	P/C

HH-400	34	83.7	0.054	0.141	—
HH-500	32	81.6	0.043	0.183	—
HH-600	30	81.2	0.033	0.199	—
HH-700	32	81.3	0.043	0.187	—

H-1000	84	81.5	0.055	0.172	—

PH-400	48	83.0	0.027	0.164	0.015
PH-500	243	82.3	0.036	0.164	0.016
PH-600	311	82.4	0.025	0.174	0.015
PH-700	674	81.6	0.035	0.166	0.024
PH-800	564	80.4	0.043	0.181	0.020
PH-900	1008	82.6	0.036	0.157	0.017

P-1000	1014	84.0	0.043	0.125	0.022

**Figure 4:**
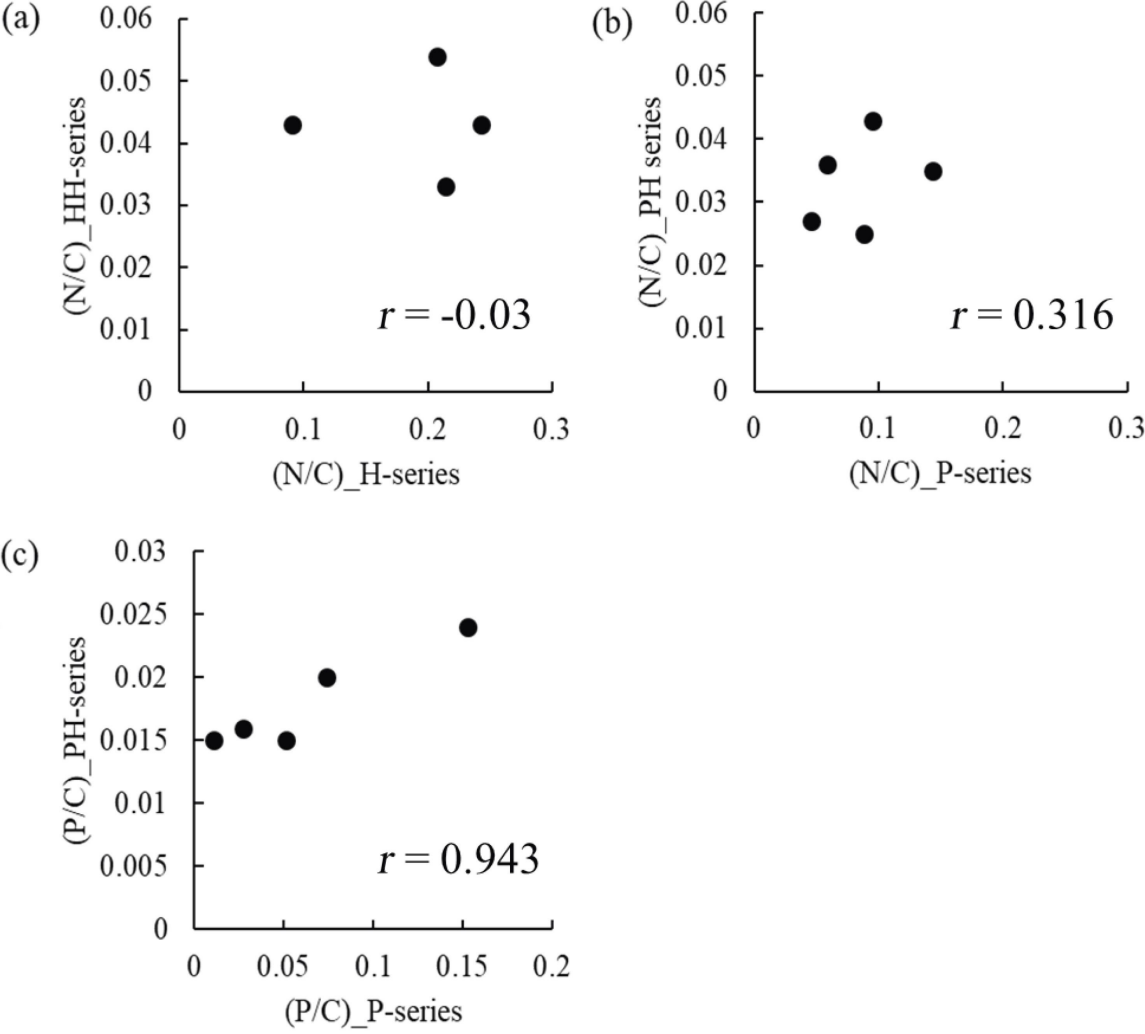
Correlations between (a) N/C ratios of HH-series carbon materials and those of H-series precursors, (b) N/C ratios of PH-series carbon materials and those of P-series precursors, (c) P/C ratios of PH-series carbon materials and those of P-series precursors. The figures show the linear correlation coefficients, *r*, calculated from least mean-square analysis of the plots.

Table 3 also lists N/C and P/C atomic ratios determined by XPS, demonstrating that for HH- and PH-series carbon materials. The former ratio showed no clear dependence on the pretreatment temperature and was in the range of 0.03–0.05. Conversely, the P/C atomic ratio of PH-series carbon materials varied in the range of 0.015–0.024, with a maximum value obtained at 700 °C. Figure 4 shows the relationship between the elemental composition of the precursors and that of the carbon materials, revealing that the N content of the precursors had no influence on that of the carbon materials, while the P content of PH-series carbon materials was positively correlated with that of P-series precursors with a linear correlation coefficient of *r* = 0.943. These results confirmed the viability of the CPAT method and demonstrated that carbon materials with a high extent of P-doping can be prepared from precursors with a high P/C atomic ratio.

**Figure 5:**
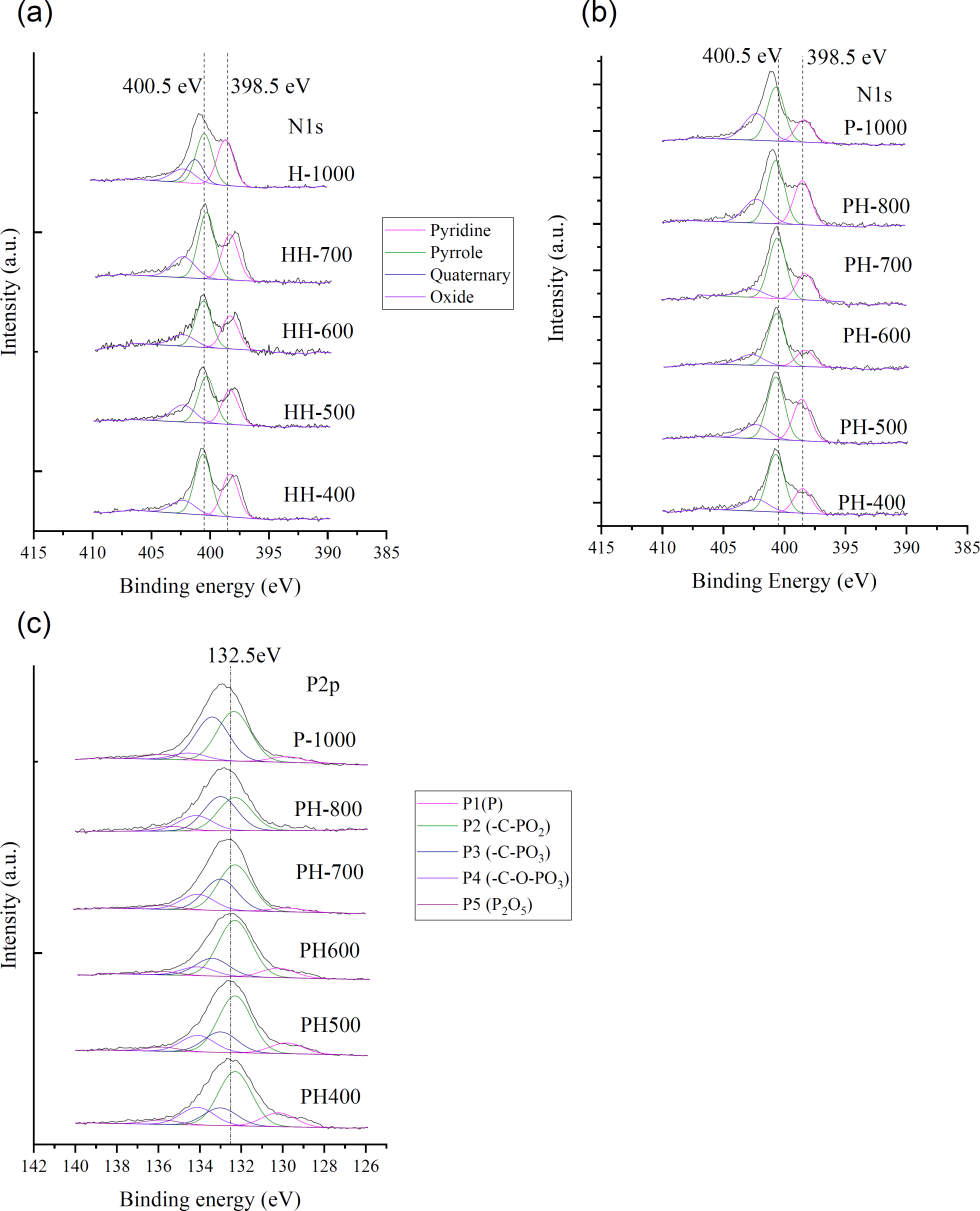
N 1s spectra of (a) HH- and (b) PH-series carbon materials. (c) P 2p spectra of PH-series carbon materials.

Figure 5a,b shows that although the N 1s spectra of both HH- and PH-series carbon materials comprise two peaks, the relative intensities of these two peaks were different, as exemplified by the spectra of H-1000 and P-1000. The shapes of the N 1s spectra of other HH- and PH-series carbon materials were similar to those of H-1000 and P-1000 spectra, respectively, and did not depend on the pretreatment temperature. The N 1s spectra were deconvoluted into the four abovementioned peaks (pyridinic, pyrrole/pyridone-type, quaternary, and oxidized N) as shown in Figure 5, with the results presented in Table 4. Notably, the spectra of PH-series carbon materials were dominated by peaks of non-pyridinic N, while those of HH-series carbon materials featured signals of pyridinic and pyrrole/pyridone-type N of comparable intensities.

**Table 4:** Distribution of N-species in HH- and PH-series carbon materials.

sample	N/C	N_pyridine_	N_pyrrol_	N_quaternary_	N_oxide_

HH-400	0.054	0.37	0.63	0.00	0.01
HH-500	0.043	0.32	0.44	0.00	0.23
HH-600	0.033	0.34	0.48	0.00	0.17
HH-700	0.043	0.32	0.47	0.00	0.21

H-1000	0.055	0.33	0.36	0.17	0.14

PH-400	0.027	0.23	0.60	0.00	0.17
PH-500	0.036	0.31	0.52	0.00	0.17
PH-600	0.025	0.20	0.62	0.00	0.18
PH-700	0.035	0.24	0.61	0.00	0.15
PH-800	0.043	0.31	0.45	0.00	0.24
PH-900	0.036	0.19	0.44	0.00	0.37

P-1000	0.043	0.20	0.47	0.00	0.33

The P 2p spectra of all PH-series carbon materials featured broad asymmetric signals at 132.5 eV (Figure 5c) that were deconvoluted into five components (Table 5). The most and second-most abundant moieties were identified as –C–PO_2_ (P2) and –C–PO_3_ (P3), respectively, and the contributions of other components (P1 (C–P): P bonded only to carbon atoms, P4 (–C–O–PO_3_: P bonded to carbon via oxygen, P5 (P_2_O_5_): P without any bonds to carbon) were found to be minor [35].

**Table 5:** Distribution of P-species in PH-series carbon materials.

sample	P/C	P1 (P) (130 eV)	P2 (–C–PO_2_) (132.5 eV)	P3 (–C–PO_3_) (133.2 eV)	P4 (–C–O–PO_3_) (134.2 eV)	P5 (P_2_O_5_) (135.6 eV)

PH-400	0.015	0.13	0.50	0.16	0.16	0.04
PH-500	0.016	0.10	0.52	0.19	0.15	0.04
PH-600	0.015	0.10	0.59	0.18	0.09	0.04
PH-700	0.024	0.04	0.46	0.31	0.16	0.04
PH-800	0.020	0.04	0.47	0.32	0.08	0.08
PH-900	0.017	0.03	0.41	0.32	0.16	0.08

P-1000	0.022	0.05	0.45	0.39	0.06	0.04

The above observations revealed that carbonization at 1000 °C attenuated the differences in the chemical states of P and N observed in the precursors. However, the effects of CPAT such as changes in the chemical states of N and the amount of P were retained.

**Figure 6:**
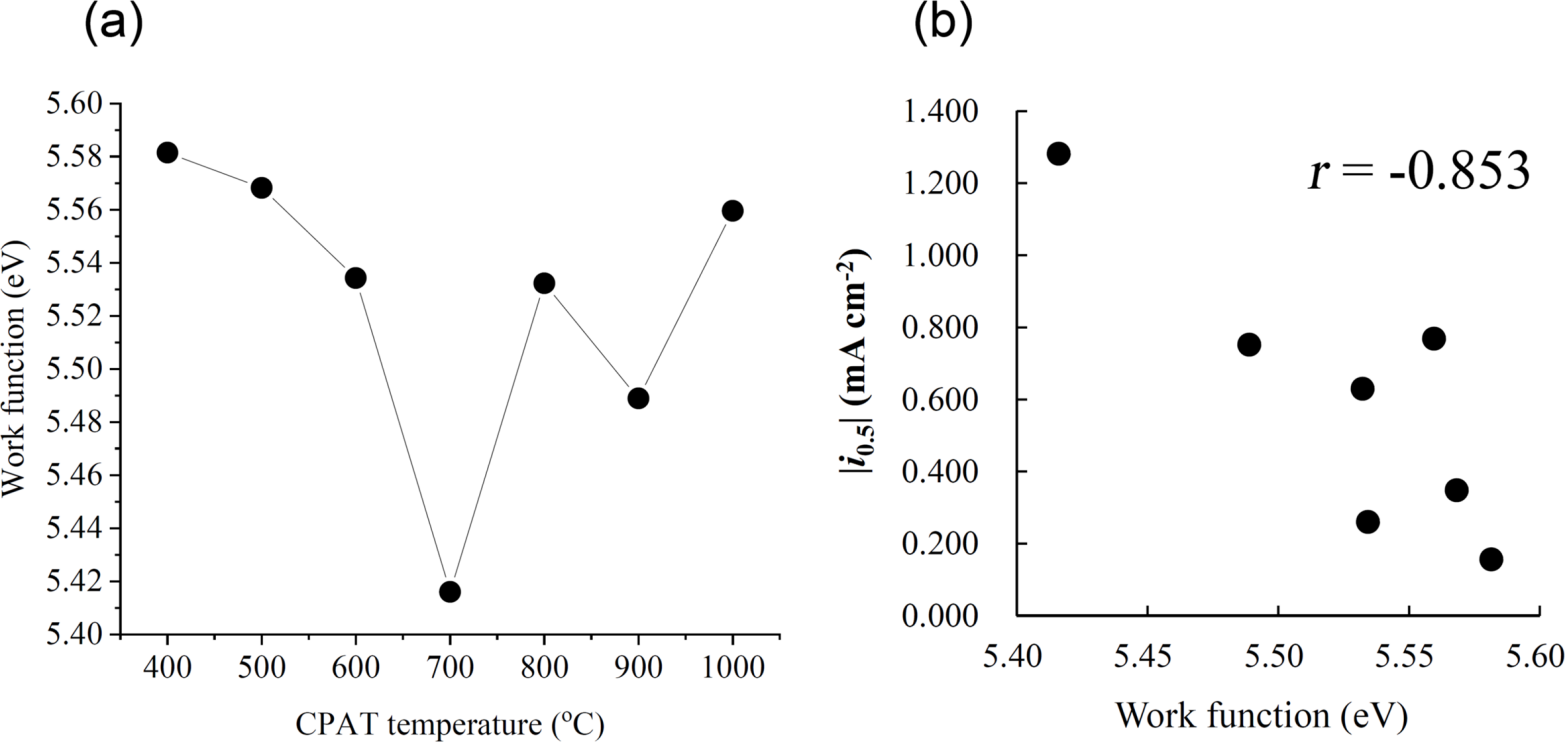
(a) Effect of CPAT temperature on the work function of PH-series carbon materials, (b) relationship between the ORR activity and the work function of carbonized samples.

The work function of PH-series carbon materials was determined by the vibration capacitance (Kelvin) method and fluctuated in the range of 5.4–5.6 eV (Figure 6a), decreasing with increasing CPAT temperature in the range of 400–700 °C and increasing with increasing CPAT temperature above 700 °C. As a result, the smallest work function was observed for PH-700. Figure 6b shows the relationship between the ORR activity and the work function. These two values exhibited a good correlation with *r* = −0.853.

### ORR activity of the carbon materials

**Figure 7:**
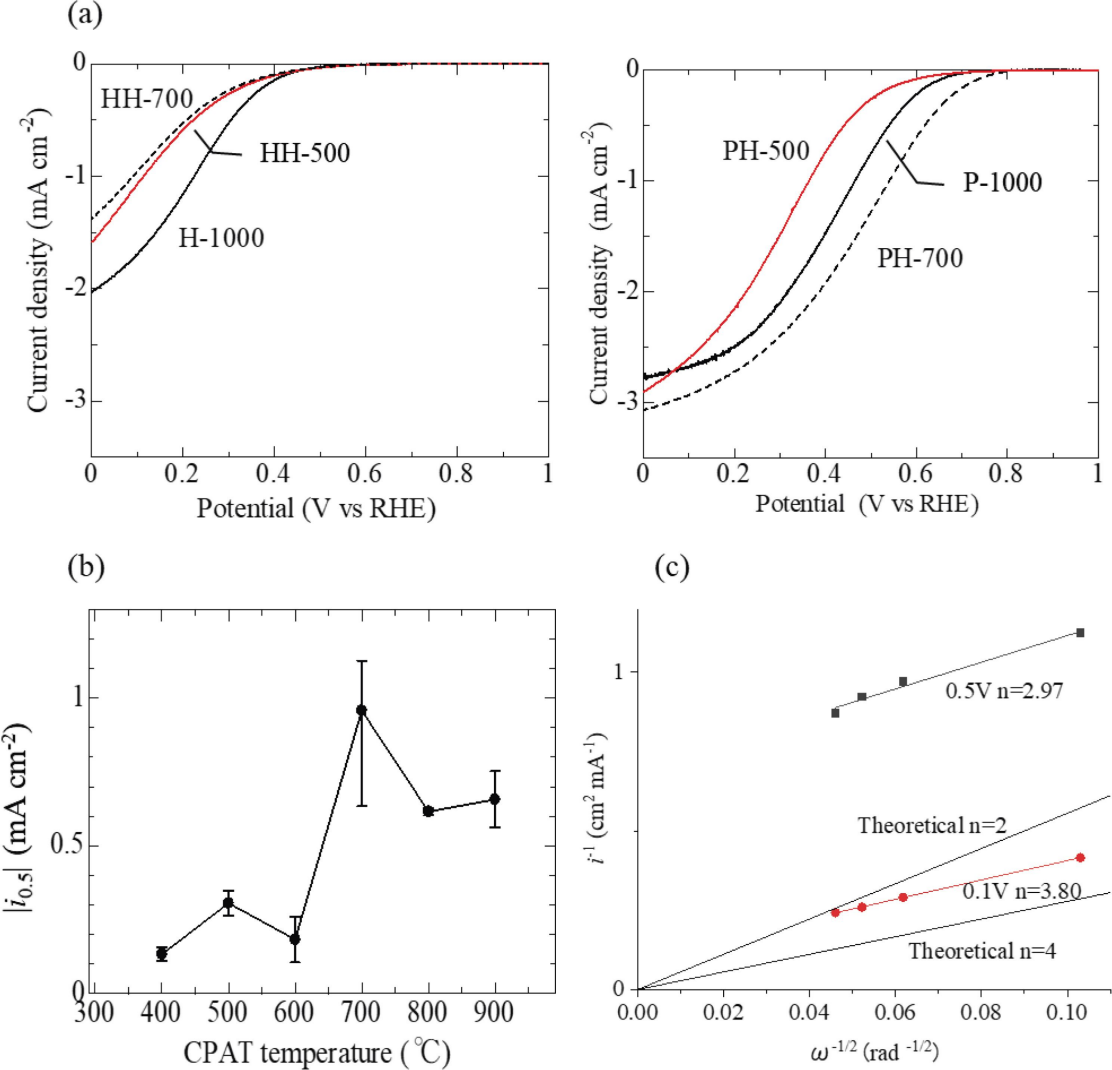
Results of ORR activity studies. (a) ORR voltammograms of HH- and PH-series carbon materials recorded in 0.5 M O_2_-saturated aqueous H_2_SO_4_. (b) Dependence of the ORR activity (represented |*i*_0.5_|) on the CPAT temperature. (c) Koutecky–Levich plots obtained for PH-700 using data acquired at 0.1 V and 0.5 V vs RHE.

Figure 7a shows representative ORR voltammograms of PH- and HH-series carbon materials recorded in O_2_-saturated aqueous H_2_SO_4_. The voltammograms of HH-500 and HH-700 were almost identical, i.e., pretreatment had no effect on ORR activity, while the voltammogram of H-1000 was different, showing a larger increase of ORR current density below 0.4 V vs RHE. At a given potential, higher current densities were observed for PH-series carbon materials than for HH-series carbon materials, which was ascribed to the influence of CPAT. Figure 7b shows the dependence of ORR activity (|*i*_0.5_|, defined as the current corresponding to a potential of 0.5 V) on the CPAT temperature. The |*i*_0.5_| values of HH-series carbon materials ranged from 0.02 to 0.03 mA·cm^−2^ regardless of the pretreatment temperature, while the |*i*_0.5_| value of PH-series carbon materials reached 0.96 mA·cm^−2^ at 700 °C and then decreased, i.e., was maximal for PH-700. Figure 7c shows Koutecky–Levich plots obtained for PH-700, revealing that at 0.5 V vs RHE, the number of electrons transferred during the ORR approximately equaled three and approached a value of four at 0.1 V vs RHE.

**Figure 8:**
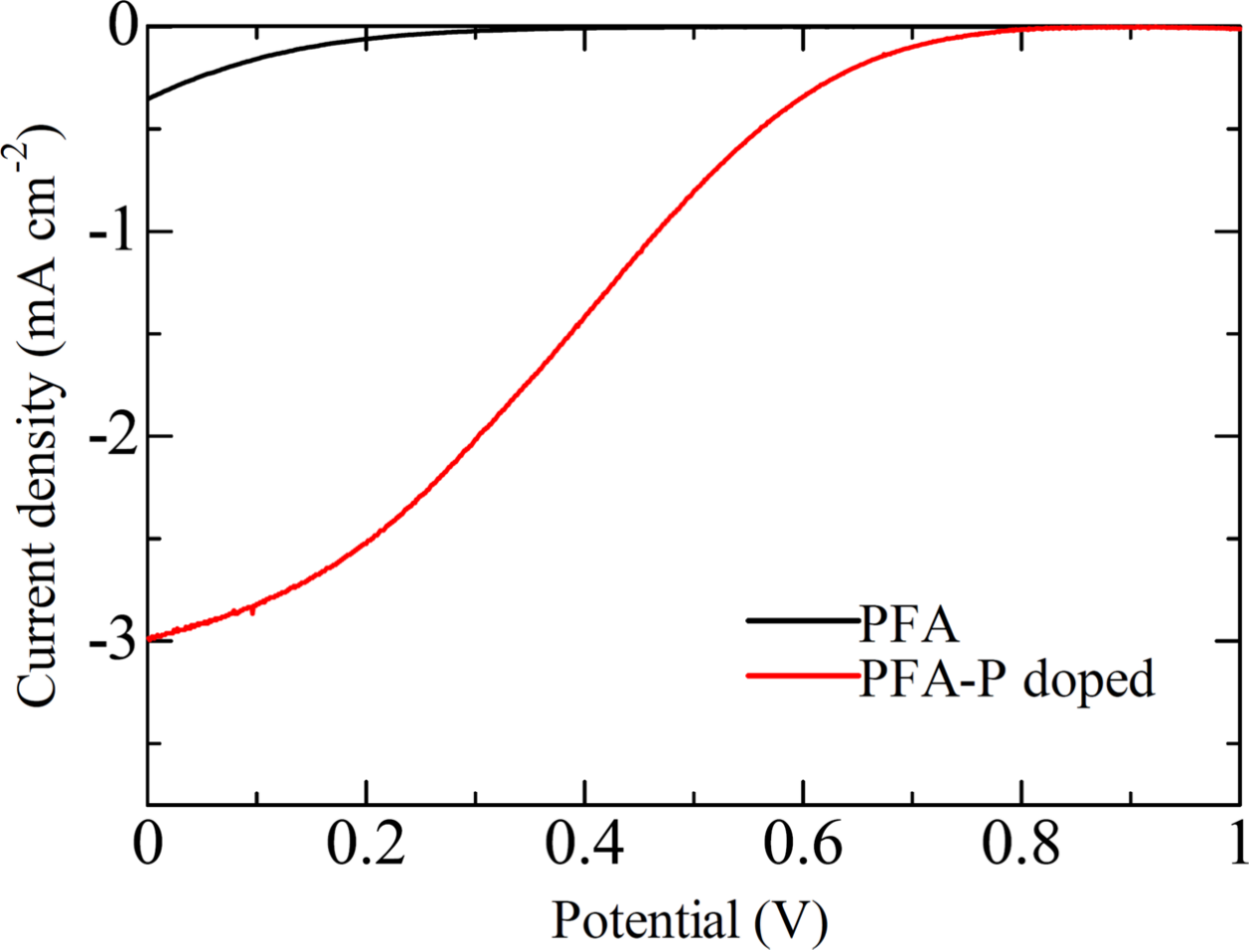
ORR voltammograms of two different PFA-derived carbon materials.

Notably, PN-doped (PH-series) carbon materials exhibited higher ORR activity than N-doped (HH-series) carbon materials. As N-free P-doped carbon materials could not be prepared from FA, a P-doped carbon material was prepared from poly(furyl alcohol) (PFA) to examine the effects of P-only doping on the ORR activity. The ORR activity of P-doped PFA-derived carbon material was higher than that of a non-doped PFA-derived carbon material (Figure 8).

**Figure 9:**
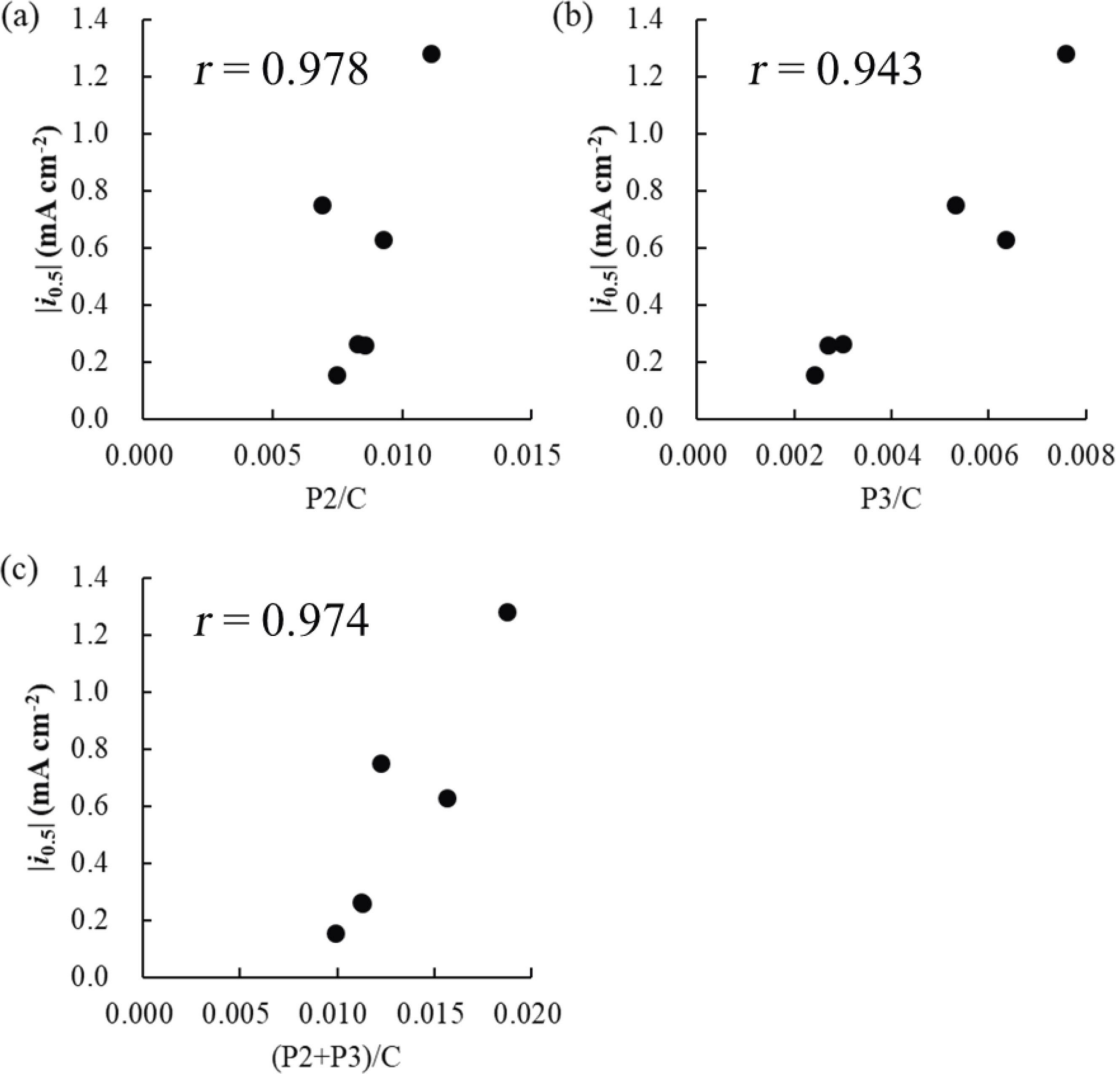
Correlation of the ORR activity (represented by *i*_0.5_) with (a) P2/C, (b) P3/C, and (c) (P2+P3)/C molar ratios.

Next, we correlated the ORR activity with BET-SSA, XPS-determined contents of N and P, and the work function. Notably, ORR activity is not fully correlated with BET-SSA (Figure S6, Supporting Information File 1) but was correlated with the relative contents of P2 (Figure 9a) and P3 (Figure 9b) species, with an even better correlation obtained between ORR activity and the sum of P2 and P3 contents (Figure 9c).

**Figure 10:**
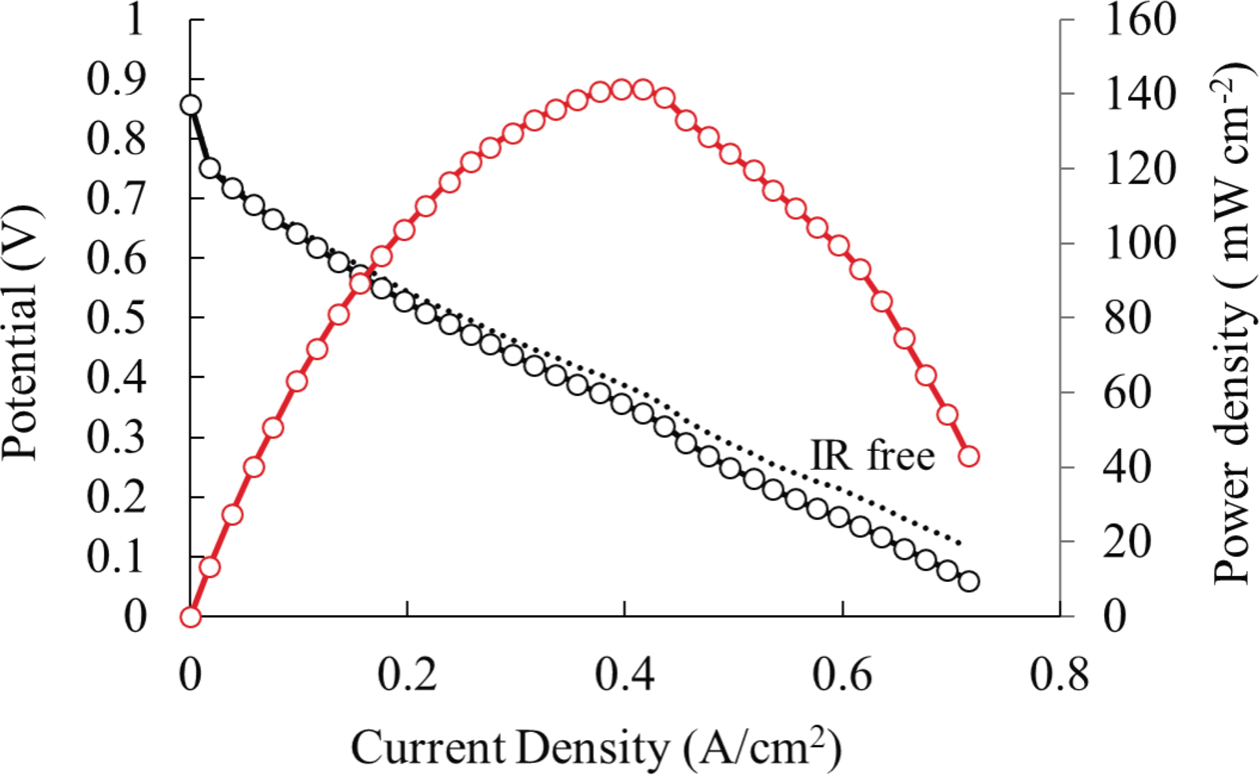
The result of a single-cell test using PH-700 as the cathode catalyst. Current density (black solid line), IR free current density (black dotted line), red solid line (power density).

Finally, the result of a single-cell test using PH-700, the catalyst with the maximum ORR activity, as the cathode catalyst and a commercial Pt/C catalyst as the anode catalyst is presented in Figure 10. The initial voltage was 0.86 V and the cell voltage decreased the current density. The red curve indicated the power density of the cell, which showed a maximum value of 141 mW/cm^2^.

## Discussion

### Doping of P into FA by CPAT

Differences between the N chemical states of P- and H-series precursors can be understood by considering the chemical interactions (possibly of the acid–base type) of PA with N atoms in FA, as evidenced by the correlation between P/C and N/C ratios (Figure 2). The N 1s spectra of P-series precursors prepared at temperatures above 400 °C had a shape different from that of the corresponding H-series precursor spectra, and 400 °C was thus taken as the onset of PA action. Interestingly, the nature of this action depended on the CPAT temperature, i.e., N loss was promoted at 400 °C, precursor co-doping with N and P atoms was promoted at 400–700 °C, and the increase of BET-SSA accompanied by the decrease of N and P content was promoted above 800 °C. As a result, maximum N and P contents were obtained at a CPAT temperature of 700 °C. This behavior agreed with the results of a previous study, where the increase of BET-SSA was shown to be accompanied by the sublimation of elemental P above 800 °C [34]. As the initial aim of CPAT was to introduce P into carbon materials rather than to increase their BET-SSAs, the temperature of 700 °C was considered to be optimal. Finally, it was concluded that PN-doped carbon precursors can be obtained by applying CPAT to other N-containing organic compounds if a proper CPAT temperature is selected.

### Chemical aspects of PN-doped carbon materials

The disappearance of the correlation between P/C and N/C ratios (Figure 2) after carbonization indicated that the latter process irreversibly destroyed interactions between N and P atoms. Additionally, the P/C ratios of PH-series carbon materials were correlated with those of P-series precursors, whereas no such correlation was observed for N/C ratios, which highlighted the need for an in-depth investigation of the chemistry involved at different preparation stages.

PN-doped (PH-series) carbon materials had a lower content of pyridinic N than HH-series carbon materials (Figure 5a,b), i.e., P-doping suppressed the formation of this type of N moieties. This behavior contradicted the results of previous studies on PN-doped carbon materials, which reported the facile formation of pyridinic N upon P-doping. For example, Gao et al. prepared a PN-doped carbon material by carbonization of an ionic liquid synthesized from *N*-methylimidazole and PA and reported the selective formation of pyridinic N due to the introduction of P [36]. Li et al. reported that a carbon material obtained by carbonization of P-doped aniline-coated single-wall carbon nanotubes was rich in pyridinic N [26], while Razmjooei et al. described the influence of P-doping on the formation of pyridinic and pyrrole-type N in N, S, P-doped carbon materials [24].

The 400.5 eV peak observed in the N 1s spectra of the PH-series carbon materials has traditionally been assigned to pyrrole/pyridone-type N. The electron configuration of pyridinic N can be described as follows: Two out of five N valence electrons are used for σ-bond formation, two more electrons form an unshared electron pair, and the remaining electron is donated to the π-electron system. Conversely, in the case of pyrrolic N, two valence electrons are used to form C–N–C σ-bonds, one electron is used to form the N–H bond, and the remaining two electrons are donated to the π-electron system. These differences in the number of electrons supplied to the π-electron system result in differences in the N 1s peak binding energies. Strelko et al. conducted quantum chemical calculations to characterize N-doped graphene, revealing that the electronic states of hydrogenated pyridinic N and the three-coordinated N located in the valley at the zigzag edge of graphene are similar to that of pyrrolic N [21].

Detailed analysis of N 1s and P 2p spectra showed that the presence of N–P bonds in carbonized products can be excluded and demonstrated that P was mainly present as –C–PO_2_. However, it was difficult to prove the presence of P–N and P=N bonds by analysis of N 1s spectra, as the similar binding energy values of N–P moieties (398.5 eV) and pyridinic N, and of N=P moieties (401.3 eV) and quaternary N made unambiguous assignments impossible [37–38]. Thus, as the N 1s spectra of PH-series carbon materials indicated the absence of N–P and N=P bonds, we concluded that these carbon materials did not contain the above moieties. Regarding P 2p spectra, the main species were identified as P2 (132.5 eV; –C–PO_2_) and P3 (133.2 eV; –C–PO_3_). As the P–N signal usually appears at 133.5 eV and overlaps with that of P3, PH-series carbon materials were concluded to contain P2 and P3 as major species and feature no N–P moieties.

### Factors determining ORR activity

As electrocatalytic ORR is a heterogeneous reaction occurring on solid surfaces, the overall catalytic activity is governed by the surface area involved in the reaction and the type and surface density of active sites. Herein, the ORR activity of PH-series carbon materials was found to be only weakly influenced by the BET-SSA values (Figure S6, Supporting Information File 1) but was rather determined by the abundances of P2 and P3 species, i.e., by the contents of P atoms directly bonded to one or two carbon atoms as shown in Figure 9.

Previously, the enhanced ORR activity of PN-doped carbon materials was ascribed to an increase of asymmetric spin density [24–25,39], electron transfer from N or P to C [36], changes of oxygen adsorption ability [26,40], and the formation of pyridinic N active sites due to P-doping [26]. In our case, the last reason, namely the formation of pyridinic N, can be ruled out, while further studies are required to confirm/disprove the influence of oxygen adsorption properties. At this point, it is worth noting that our previous investigations of the relationship between the ORR activity and oxygen adsorption properties of warped graphitic layers (obtained by oxidative heat treatment of fullerene extraction residues) demonstrated that these two parameters are well correlated [41].

The ORR activity was well correlated with the work function (Figure 6b), which represents the energy of the Fermi level with respect to that of the vacuum level that would be brought by the introduction of P2 and P3 species, showing good correlation as discussed above. Several reports on the relationship between the work function of cathode catalysts and their ORR activity demonstrated that the former parameter strongly influences the latter and affects electron transfer in elementary reaction steps. As the Fermi level is the highest-energy electronic level of a given solid, the ORR reaction proceeds spontaneously when this level exceeds the ORR standard potential of 5.6 eV [42–44]. As shown in Figure 6a, PH-series carbon materials had work functions of less than 5.6 eV and could therefore spontaneously promote the ORR. The two structural features of PN-doped carbon materials, i.e., the presences of particular P-containing species and the warped graphitic layers, should be the important factors determining ORR activity through facilitating O_2_ adsorption and/or electron transfer at the catalyst surface.

## Conclusion

Herein, we applied controlled phosphoric acid treatment (CPAT) of folic acid (FA) to prepare P-doped precursors, which were then carbonized to afford PN-doped carbon materials as oxygen reduction reaction (ORR) catalysts. Essentially, FA was heated in the presence of phosphoric acid at an optimal temperature of 700 °C to maximize the P content of precursors before the occurrence of chemical activation. The P/C ratio of precursors was found to be positively correlated with that of the corresponding carbon materials and carbon ORR activity. In contrast to previous studies, where ORR activity has been largely attributed to the presence of active sites based on pyridinic N, the enhanced ORR activity of our carbon materials was ascribed to the presence of –C–PO_2_ and C–PO_3_ moieties. Moreover, this activity increased with decreasing work function of the carbon materials. Given that an optimal treatment temperature is selected, we believe that the CPAT technique can be applied to all types of N-containing compounds, e.g., naturally occurring ones. However, fundamental studies on the kinetics and mechanisms of ORR activity enhancement induced by PN-doping are required to clarify the remaining questions and will be conducted in due course.

## Experimental

### CPAT

N- and P-containing precursors were prepared by heating FA in the presence of PA. Typically, FA (1 g; Wako, Wako Special Grade) was ground with ethanolic PA (85 wt %, 1 g; Wako, Wako Special Grade) using a mortar and pestle, and the obtained mixture was placed in a furnace, heated to 400–800 °C in a flow of N_2_ at a rate of 50 °C·min^−1^, and then held at this temperature for 1 h. The carbonized samples were then pulverized at 650 rpm for 50 min using a planetary ball mill (P-7, Fritsch), sieved to retrieve particles smaller than 106 μm in diameter, vigorously stirred in deionized water at 80 °C for 1 h, and dried to obtain P-T specimens (T = CPAT temperature). Controls were prepared in the same manner without the addition of PA and were referred to as H-T (T = pretreatment temperature).

### Carbonization

The doped and control precursors were carbonized at 1000 °C for 1 h in a stream of N_2_ to afford PH-T and HH-T specimens, respectively (T = treatment temperature). Moreover, carbon materials were also prepared by directly heating FA or PA-FA mixtures to 1000 °C (H-1000 and P-1000 samples, respectively). To study the influence of P-only doping, the above carbonization procedure was applied to poly(furfuryl alcohol) (PFA). Two types of PFA-based carbon materials were prepared by using hydrochloric acid or phosphoric acid as polymerization initiators (non-doped and P-doped PFA carbon materials, respectively).

### Electrochemical methods

The ORR activity of carbon materials was probed by rotating disk electrode voltammetry. The working electrode was prepared by loading the catalyst (200 μg·cm^−2^) on a glassy carbon disk electrode. The carbon ink was prepared in the following manner: 2.5 mg of the prepared sample was mixed with 25 μL of Nafion solution (5% solution of lower aliphatic alcohols, Aldrich), 75 μL of ethanol (99.5%, Wako Pure Chemicals, Co. Ltd.) and 75 μL of ultrapure water in a plastic conical vial (1.5 mL). The working electrode was a 4 mm diameter glass-like carbon electrode (BAS Inc.). The carbon ink (1.78 μL) was pasted onto the whole area of the glass-like carbon electrode (catalyst loading is 200 μg·cm^−2^). A reversible hydrogen electrode (RHE) and a glassy carbon plate were employed as reference and counter electrodes, respectively. The electrolyte was a 0.5 M solution of H_2_SO_4_ in deionized water. Prior to the measurements, dissolved oxygen in the acid solution was purged by bubbling nitrogen gas. Cyclic voltammetry measurements were performed by sweeping the potential between 0.0 V and 1.0 V vs RHE at 50 mV/s for five cycles with a potentiostat (ALS 2323, BAS Inc.). Net ORR voltammograms were obtained as the difference between linear sweep voltammograms recorded at 1500 rpm in O_2_-saturated and N_2_-saturated electrolytes (RRDE-3A, BAS Inc.). Koutecky–Levich analysis was conducted for a selected sample by taking *D*_O2_ = 1.40 × 10^−5^ cm^2^·s^−1^ and *v* = 1.00 × 10^−2^ cm^2^·s^−1^ [45]. The oxygen concentration was determined using an optical oxygen meter (FireStingO_2_, Pyro Science GmbH) as *C*_O2_ = 1.20 × 10^−6^ mol·cm^−3^. Prior to the tests of the prepared samples, we evaluated Pt/C (IFPC40, ISHIFUKU Metal Industry Co., Ltd.), and the onset potential of Pt/C was 0.96 V.

The membrane–electrode assembly was fabricated as follows: The catalyst ink, i.e., the dispersion of the catalyst in Nafion solution ((5 wt % solution of lower aliphatic alcohols, Aldrich), ionomer/catalyst weight ratio ≈ 0.7:1) was sprayed onto a diffusion layer (29BC, SGL CARBON GmbH). A Pt/C catalyst was used as the anode (catalyst loading = 0.3 mg·cm^−2^), and PH-700 was used as the cathode (catalyst loading = 3.5 mg·cm^−2^). A 5 cm^2^ cell was used for fuel-cell testing. Polarization curves were obtained at a cell temperature of 80 °C, a back pressure of 200 kPa, and a reactant gas relative humidity of 100% using H_2_ (1 L·min^−1^) and O_2_ (1 L·min^−1^) as anode and cathode gases, respectively.

### Characterization techniques

The Brunauer–Emmett–Teller (BET) surface area was evaluated by N_2_ adsorption measurements (BELSORP Max, Microtrac BEL). Samples were placed in a tube and degassed at 200 °C for 2 h under dynamic vacuum conditions. C 1s, N 1s, O 1s, and P 2p core-level X-ray photoelectron spectra were recorded using Mg Kα radiation (Kratos AXIS-NOVA, Shimadzu Corp.). Generally, the charge-up shift correction was performed by setting the C 1s peak binding energy to 284.5 eV. Charge-up corrections for PA-400, 500, and 600 were performed by bringing these samples into contact with In foil and setting the In 3d peak binding energy to 451.4 eV. Work functions were measured under N_2_ by a vibration capacity electrometer (DCU series10, KP Technology).

## Supporting Information

File 1 Additional experimental data.
