# Supplementary material for: Synthesis of P- and N-doped carbon catalysts for the oxygen reduction reaction via controlled phosphoric acid treatment of folic acid
Source: Beilstein J Nanotechnol. 2019 Jul 25;10:1497–510. doi: 10.3762/bjnano.10.148 (PMC6664403; doi:10.3762/bjnano.10.148)
Supplement: File 1 — Additional experimental data. [file Beilstein_J_Nanotechnol-10-1497-s001.pdf]

BEILSTEIN JOURNAL OF NANOTECHNOLOGY

Supporting Information

for

Synthesis of P- and N-doped carbon catalysts for the oxygen reduction reaction via controlled phosphoric acid treatment of folic acid

Rieko Kobayashi, Takafumi Ishii, Yasuo Imashiro and Jun-ichi Ozaki

Beilstein J. Nanotechnol. **2019,** *10,* 1497–1510. doi:10.3762/bjnano.10.148

Additional experimental data

License and Terms: This is a supporting information file under the terms of the Creative Commons Attribution License (http://creativecommons.org/licenses/by/4.0). Please note that the reuse, redistribution and reproduction in particular requires that the authors and source are credited.
The license is subject to the *Beilstein Journal of Nanotechnology* terms and conditions: (https://www.beilstein-journals.org/bjnano)

Pore structures of the precursors

The pore structures of the precursors prepared at different heat-treatment temperatures are shown in Figure S1. The N_2-adsorption isotherms of H-series samples, shown in Figure S1a-1, commonly showed a small adsorption at the lowest relative pressure region followed by a gentle increase in the middle relative pressure region and a steep increase at near the relative pressure of unity. The volume of the adsorbed N_2 increased depending on the pretreatment temperature. P-series samples (Figure S1b-1) showed a different temperature-dependence of the isotherms, particularly when they were prepared above 700 °C, which induced an abrupt increase in the volume of N_2 adsorbed. Another large increase in the N_2 volume adsorbed was observed when CPAT was conducted at 1000 °C. The micropore size distribution curves for H-series and P-series precursors are presented in Figure S1a-2 and Figure S1b-2, respectively. The pore size distribution curve of H-1000 indicated the formation of micropores with uniform pore size. In the case of P-series, the development of uniform micropores were observed for the precursors prepared at higher than

800 °C. Note that the ordinate scales of the graphs for H-series and P-series are different.

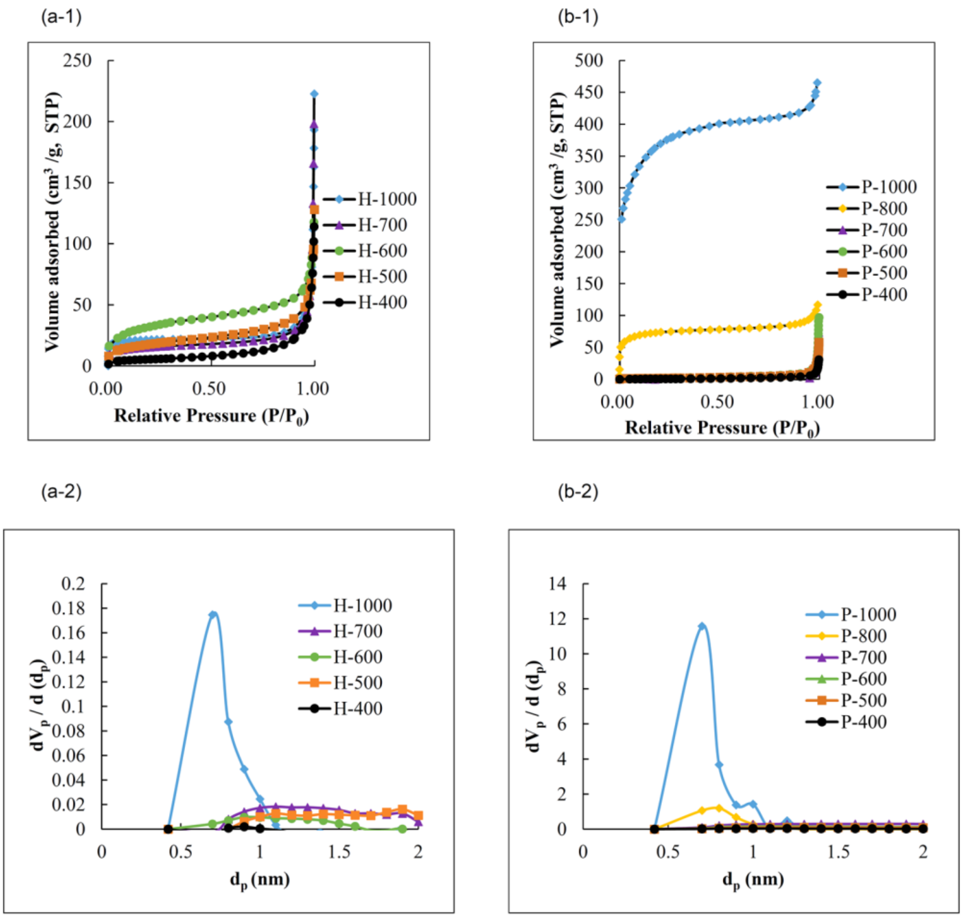

Figure S1: N_2 adsorption isotherms of H-series (a-1), P-series (b-1), and micropore size distribution by MP method, H-series (a-2), P-series (b-2). The isotherms were obtained at the liquid nitrogen with an automatic apparatus (BELSORP mini, MicrotracBEL Corp.). Before the measurements, the samples were evacuated at 200 °C for 2 h.

Morphology of the samples

Figure S2 shows the SEM images of the prepared carbon materials. Irregularly shaped particles were observed because these particles were obtained by ball-milling. The samples treated with phosphoric acid, P-1000 and PH-1000 seemed to have larger particles than H-series samples. SEM was operated at an acceleration voltage of 5 kV (JSM-6700FS, JEOL) and carried out for the ground samples placed on conductive carbon tape.

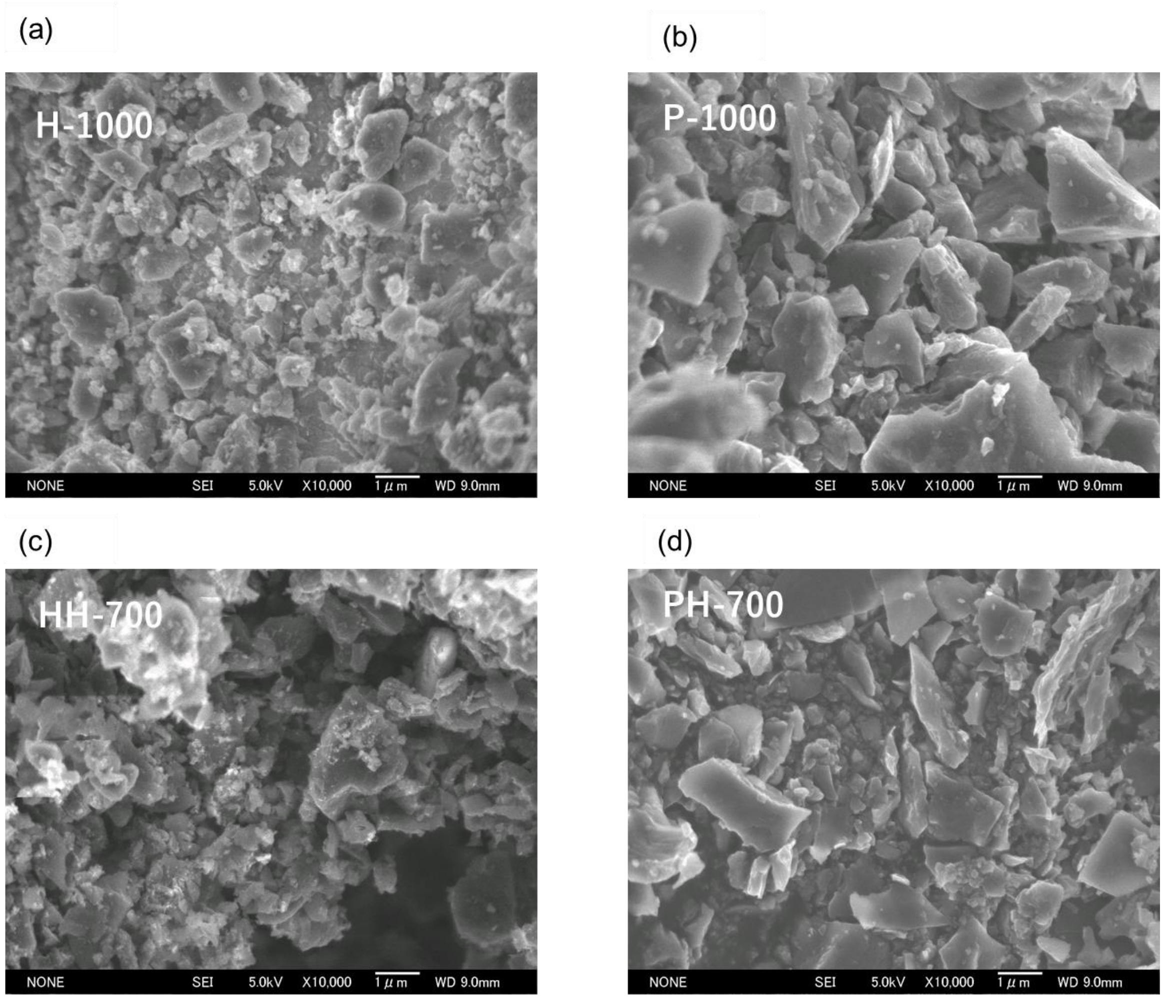

Figure S2: Scanning electron microscopic (SEM) images of (a) H-1000, (b) P-1000, (c) HH-700, (d) PH-700.

Raman spectra of the precursors

Raman spectroscopy was applied to the precursors to obtain information about the structural changes during the pretreatments. The results are presented in Figure S3. The precursors prepared at 400 °C, H-400 and P-400, showed somewhat different spectral shapes with additional peaks at lower Raman shifts,

1530 cm^{-1} and 1544 cm^{-1} for H400, 1241 cm^{-1} and 1250 cm^{-1} for P-400, than the G-band (1585 cm^{-1}) or D-band (1362 cm^{-1}), respectively. Increasing the pretreatment temperature resulted in giving uniform spectra consisting of G- and D-bands. Raman spectra of the prepared carbon catalysts were obtained using a Raman spectrometer (Nicolet Almega XR, Thermo Fisher Scientific, Inc.) equipped with a 532 nm Nd:YAG laser.

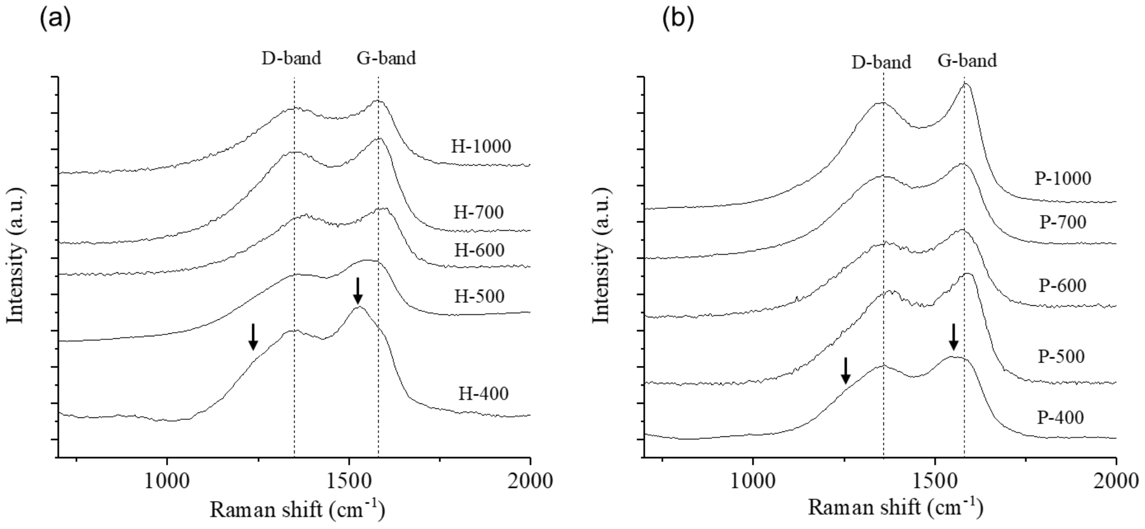

Figure S3: Raman spectra of the precursors: (a) H-series and (b) P-series.

ORR activities of the precursors

ORR voltammograms of selected precursors are presented in Figure S4. The ORR activity of H-series increased when the treatment temperature increased from 500 to 700 °C. However, it stayed nearly the same ORR activity by raising the temperature to 1000 °C. Different temperature-dependence of the ORR activity was observed for P-series. The activity increased when the temperature increased from 500 to 700 °C and a remarkably large increase was observed when the temperature changed from 700 °C to 1000 °C.

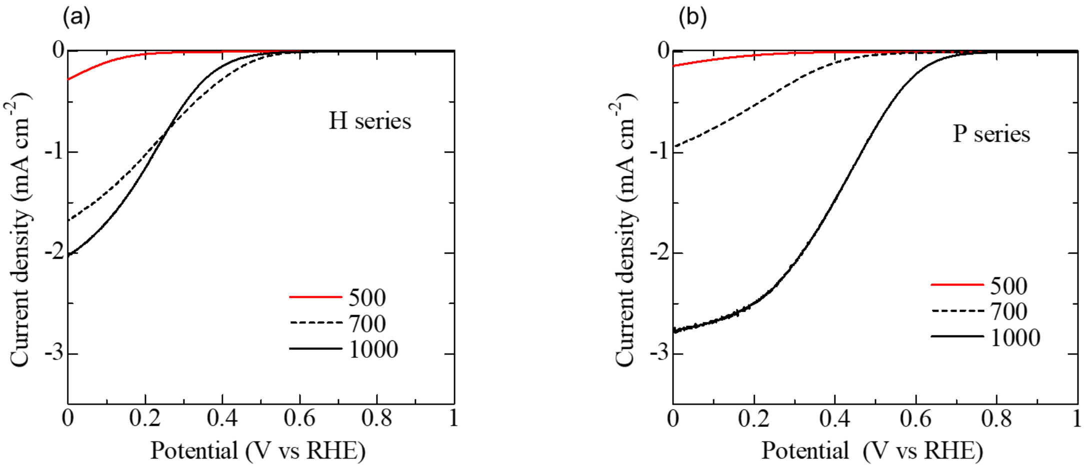

Figure S4: ORR voltammograms of H series (a) and P series (b), recorded in 0.5 M O_2-saturated aqueous H_2SO_4.

Pore structures of carbon materials

Figured S5 shows the N_2 adsorption isotherms and the micropore size distribution curves of the carbon materials prepared in the present study. The isotherms of HH-series (Figure S5a-1) completely overlapped regardless of the pretreatment temperature. Hence, the micropore size distribution of the carbon materials belonging to HH-series were the same (Figure S5a-2). On the other hand, the isotherms of PH-series carbon materials depended on the pretreatment (CPAT) temperature (Figure S5b-1). Accordingly, the micropore size distribution changed as shown in Figure S5b-2.

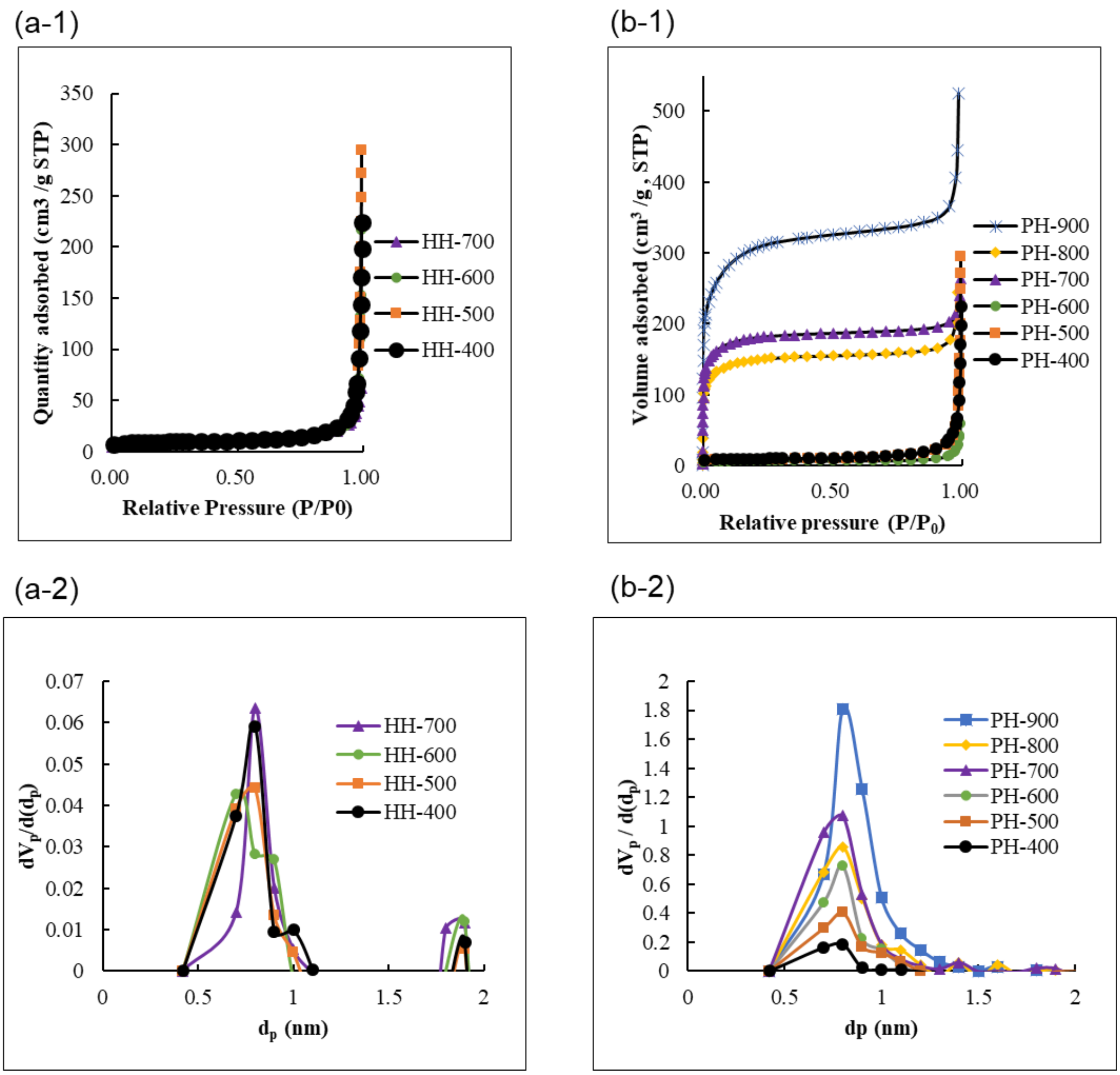

Figure S5: N_2 adsorption isotherms of HH-series (a-1), PH-series (b-1), micropore size distribution by MP-method, HH-series (a-2), PH-series (b-2).

Influence of surface area on ORR activity

In general, catalytic activity of heterogenous catalyst depends on the surface area. Figure S6 shows a relation between the ORR activity of the carbon

materials prepared in the present study and the BET surface area. A weak correlation was observed for the samples with lower BET-SSAs; however, such relation was not observed for the carbon materials with higher ORR activities. This result concluded that the higher ORR activities observed for PH-series carbon materials do not originate from the development of the surface area but also from other factors. We assumed the presence of $–C–PO_2$ and $–C–PO_3$ is responsible for the ORR activities.

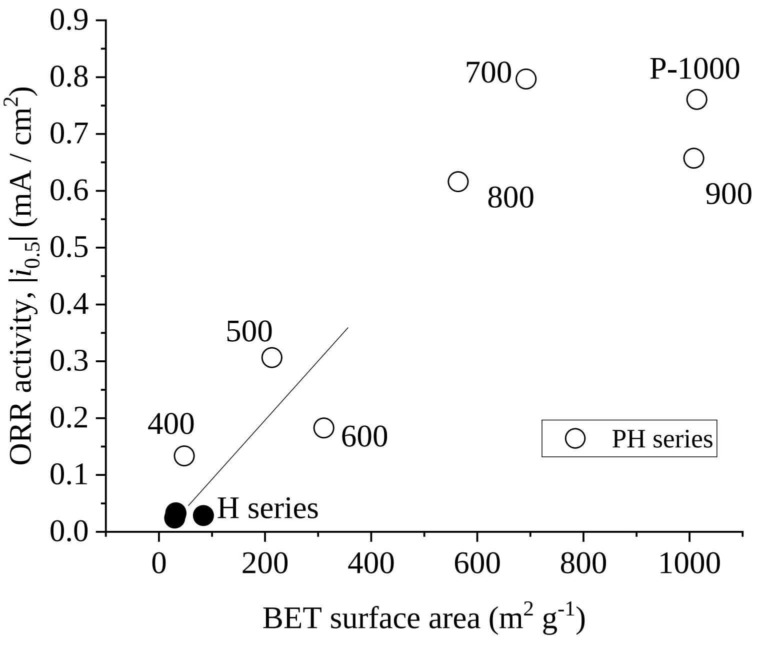

Figure S6: A relation between the ORR activity and the BET-SSA of the carbon materials prepared in the present study.

Comparison of ORR activity of carbon materials with Pt/C

Figure S7 compares the ORR activity of the carbon materials prepared in the present study with the commercial Pt/C catalyst in an acidic electrolyte. The catalyst with highest ORR activity in the present study, PH-700, showed a voltammogram close to that of Pt/C, even the carbon did not include any metal species promoting ORR activity. The Pt/C sample contained 40 wt % Pt (IFPC40, ISHIFUKU Metal Industry Co., Ltd.).

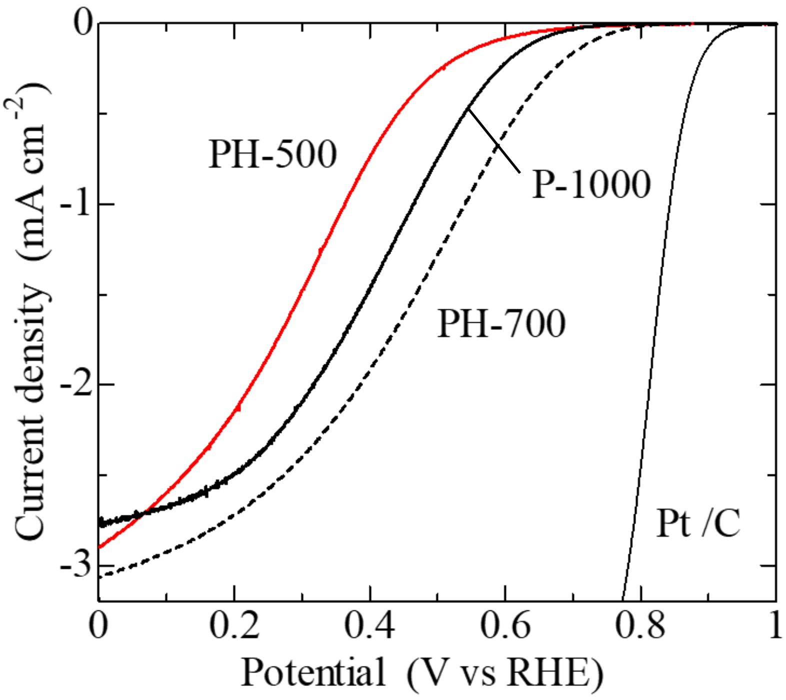

Figure S7: Results of ORR activity studies, ORR voltammograms of carbonized CPAT samples at pH 0 (0.5 M H_2SO_4) with Pt/C.
